# Molecular characterization of cyclophilin A-like protein from *Piriformospora indica* for its potential role to abiotic stress tolerance in *E. coli*

**DOI:** 10.1186/1756-0500-6-555

**Published:** 2013-12-23

**Authors:** Dipesh Kumar Trivedi, Mohammed Wahid Ansari, Tanima Dutta, Prabhjeet Singh, Narendra Tuteja

**Affiliations:** 1 Plant Molecular Biology Group, International Centre for Genetic Engineering and Biotechnology (ICGEB), Aruna Asaf Ali Marg, New Delhi 110 067, India; 2 Department of Biotechnology, Guru Nanak Dev University, Amritsar 143 005, Punjab India

## Abstract

**Background:**

Cyclophilins (CyP), conserved in all genera, are known to have regulatory responses of various cellular processes including stress tolerance. Interestingly, CyP has a crucial role as peptidyl-prolyl *cis*–*trans* isomerases (PPIases). Our earlier *in silico* based approach resulted into the identification of cyclophilin family from rice, *Arabidopsis* and yeast. In our recent report, we discovered a new OsCYP-25 from rice. Here, we identified a novel cyclophylin A-like protein (PiCyP) from *Piriformospora indica* which is responsible for abiotic stress tolerance in *E. coli*.

**Results:**

Cyclophylin A-like protein (CyPA) (accession number GQ214003) was selected from cDNA library. The genomic organization CyPA revealed a 1304 bp of *CyPA* in *P. indica* genome, showing 10 exons and 9 introns. Further, *CyPA* was evident in PCR with gDNA and cDNA and Southern blot analysis. The phylogenetic examination of CyPA of *P. indica* showed that it is closed to human cyclophilin. The uniqueness of PiCyPA protein was apparent in western blot study. Kinetics of purified PiCyPA protein for its PPIas activity was determined via first order rate constant (0.104 s^-1^) in the presence of 1 μg of PiCyPA, with increasing PiCyPA concentration, in the presence of cyclosporin A (CsA) and the inhibition constant (4.435 nM) of CsA for inhibition of PiCyPA. The differential response of *E. coli* harbouring pET28a-*PiCypA* was observed for their different degree of tolerance to different abiotic stresses as compared to empty pET28a vector.

**Conclusions:**

Overexpression of PiCyPA protein *E. coli* cells confer enhanced tolerance to wide range of abiotic stresses. Thus, this study provides the significance of PiCypA as a molecular chaperone which advanced cellular stress responses of *E. coli* cells under adverse conditions, and it, furthermore, confirms the mounting the sustainability of *E. coli* for exploitation in recombinant proteins production. Additionally, the *PiCyPA* gene cooperates substantial functions in cellular network of stress tolerance mechanism, essentially required for various developmental stages, and might be a potential paramount candidate for crop improvement and its sustainable production under adverse conditions.

## Background

Worldwide salinity problems affect approximately 3,230,000 km^2^ area of land that threatens plant growth and agricultural productivity [1]. A plant-root-colonizing basidiomycete fungus *Piriformospora indica* (*P.indica*) was discovered in the Indian Thar desert that has shown to provide strong growth-promoting activity during its symbiosis with a broad spectrum of plants [2]. Salt stress studies have indicated promising effect of *P. indica* in barley [3]. It was shown that *P. indica* leads to early flowering, higher biomass and altered secondary metabolites of the medicinal plant, *Coleus forskohlii*[4]. Cyclophilins (CyP) are widely distributed and abundantly found proteins in eukaryotic and prokaryotic systems, which present in cytosol as well as nucleus [5]. Extensive studies of various model organisms have suggested that cyclophilins are involved directly or indirectly in wide range of cellular processes including cell division [6], transcriptional regulation [7], protein trafficking [8], cell signaling [9,10], pre-mRNA splicing [11], molecular chaperone [12,13] and stress tolerance [14,15]. They are known to possess enzymatic activity in the form of peptidyl-prolyl *cis*-*trans* isomerase (PPIase) catalysis, a reaction thought to be involved in the late stages of protein folding [16,17]. Molecular mechanism of PPIase activity in human T-cells has already been characterized structurally as well as biochemically [18]. CyPA from human T-cell has high affinity for the immune-suppressive drug Cyclosporin A (CsA) [19] and its PPIase activity can be totally inhibited by CsA. CyPA has been shown to interact with Calcineurin directly and modulating the Ca^+2^ signaling in human T cells [20], which is a primary signaling molecule in majority of the cellular events and responses. In plants, CyPA was involved in signal transduction mechanism of regulation of various abiotic stresses via phosphoprotein cascade, Ca^+2^ and other secondary signaling molecules [21]. In our recent report, we identified a new class of cyclophilin OsCyP-25 (LOC_Os09 g39780) from rice (*Oryza sativa* L.), which was upregulated in response to different abiotic stresses *viz*., salinity, cold, heat and drought [22]. In the present study, we have shown the cloning, expression, purification and crystallization of CyPA homologue from *Piriformospora indica* (PiCyPA) to understand the molecular mechanism(s) involved cell signalling network during various stress response and its potential role in providing stress tolerance both in eukaryotes and prokaryotes.

## Results

### Identification, genomic organization, isolation and confirmation of a novel cyclophilin from *P. indica*

**Figure 1:**
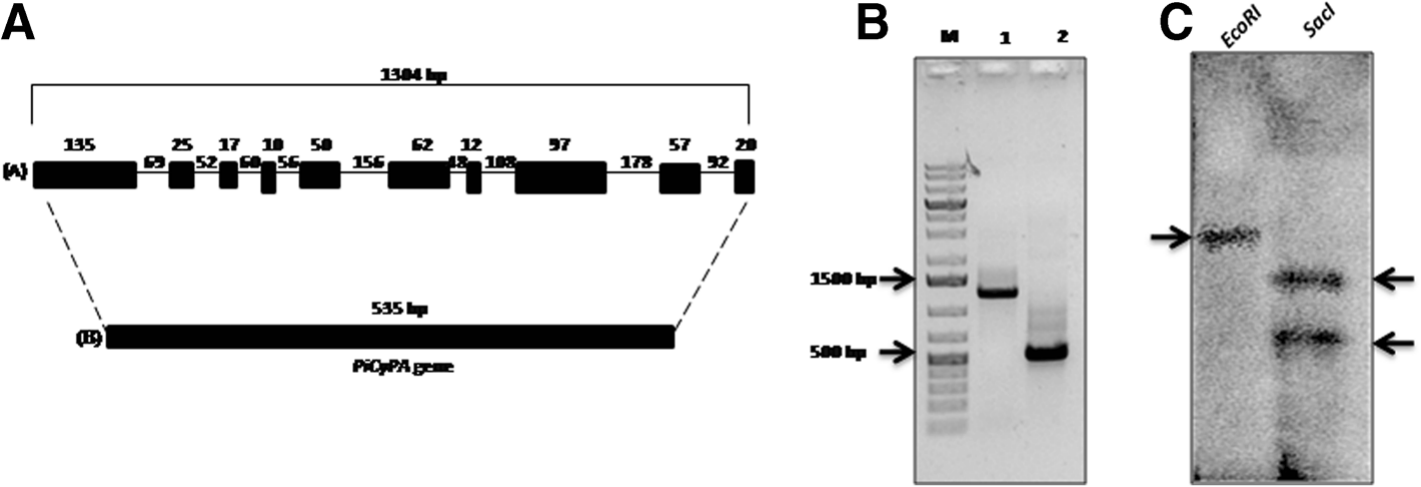
**Gnomic organization**, **PCR**- **and Southern**-**profile of *CyPA *from *P. indica*.** Genomic organization of *CyPA* gene from *P. indica* containing 10 exons and 9 introns. Introns spliced out and exons joined together and form 495 bp *PiCyPA* gene **(A)**. Genomic organization of *PiCyPA* gene confirmed by PCR, lane 1 showing PCR with genomic DNA and lane 2 showing PCR with cDNA **(B)**. Southern blotting confirmed single copy no of *PiCyPA* gene in lane 1 EcoRI used which is zero cutter and in lane 2 SacI used which is single cutter of *PiCyPA* gene **(C)**.

Cyclophylin A-like protein (CyPA) (accession number GQ214003) was selected from cDNA library for further study. Additionally, the genomic organization *CyPA* gene has been identified by using genomic sequence available on NCBI (http://www.ncbi.nlm.nih.gov) which shows that *CyPA* gene (1304 bp) in *P. indica* genome revealed 10 exons (ranged from 12–135 bp) and 9 introns (varied from 4–178 bp). Introns spliced out sequence i.e. exons sequence which stick together leading to the formation of *CyPA* gene (535 bp) (Figure 1A). Further, genomic organization of *CyPA* gene was evident from PCR amplification with *P. indica* gDNA and cDNA as a template using primers (forward: 5` CTCGAGCATATGTCCCAGCCCAACGTCTACTTTG 3` and reverse: 5`-GAATTCTTAGACAGTGCCAGACGCAGTAATCTTG 3`), displaying a band of 1304 bp and 535 bp size (Figure 1B). We have also identified the copy number(s) of *CyPA*-like gene in *P. indica* genome by Southern blotting. There was single gene copy of CyPA-like gene in *P. indica* genome, which resulted in Lane 1 by zero cutters EcoRI and within Lane 2 via single cutter SacI (Figure 1C).

### Protein alignment and phylogenetic analysis

**Figure 2:**
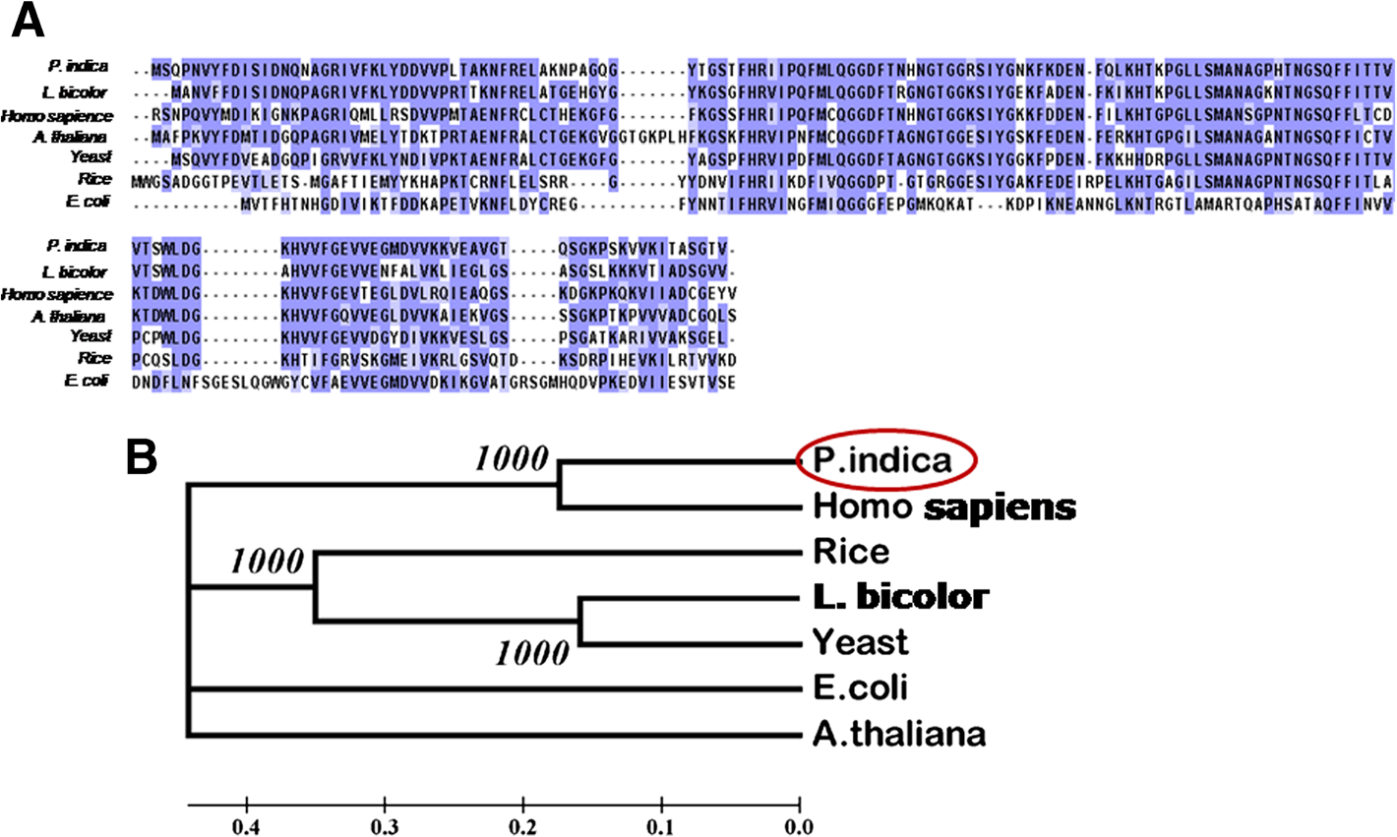
**CyPA protein sequence alignment and phylogenetic analysis.** Amino acid alignment of *CyPA* gene from *P. indica* with *L. bicolor*, *Homo sapiens*, *Arabidopsis thaliana*, yeast, rice and *E. coli* and its comparison with cyclophilin family. The highly conserved regions of the alignments have been shaded **(A)**. Phylogenetic analysis of *P. indica*, *L. bicolor*, *Homo sapiens*, *Arabidopsis thaliana*, yeast, rice and *E. coli* cyclophilin genes was constructed to examine the relationship between the cyclophilin genes in the genomes. The tree was created via neighbor-joining method using 1000 bootstrap replicates. CyP of *P. indica* is closely related to human cyclophilin in respect to high bootstrap value **(B)**.

The bioinformatic analysis of *CyPA*-like gene from *P. indica* was performed. Protein sequence of *CyPA*-like gene from *P. indica* and other organisms such as *L. bicolor*, *Homo sapiens*, *Arabidopsis thaliana*, yeast, rice and *E. coli* were aligned using ClustalW using default parameters. The comparative study of amino acid sequences of PiCyPA was performed using the UniProt BlastP Service (http://www.uniprot.org/blast/) which revealed 73, 76, 73, 62 and 38% similarity in *L. bicolor*, yeast, *Arabidopsis*, rice and *E. coli* (Table 1). We found *PiCyPA*-like gene showing high sequence similarities with cyclophilin representatives from other organisms as shown in Figure 2A. The phylogenetic analysis was also performed and we found *CyPA* of *P. indica* is closely related to human cyclophilin in respect to high bootstrap value (Figure 2B).

**Table 1:** **Percentage of similarity between CyPA of *P. indica *** **and related cyclophilins from other species of fungi**, **plant and bacteria**

**Species**	**UniProt Acc. No.**	**Gene name**	**Subcellular location**	**% similarity**	**Putitative length**	**Putitative function**
*L. bicolor*	DS547107	*CPH*	Cytosol	73%	162	Peptidyl-prolyl *cis*-*trans* isomerase
Yeast	YDR155C	*CPR1*	Cytosol	76%	163	Peptidyl-prolyl *cis*-*trans* isomerase
*Arabidopsis*	AT4G38740	*CyPROC1*	Cytosol	73%	172	Peptidyl-prolyl *cis*-*trans* isomerase
Rice	LOC_Os05g01270	CyP	Thylakoid/Plastid	62%	251	Peptidyl-prolyl *cis*-*trans* isomerase
*E. coli*	NC_007946.1	CyP B	Cellular	38%	164	Peptidyl-prolyl *cis*-*trans* isomerase

### Cloning and characterization of *PiCyPA*

**Figure 3:**
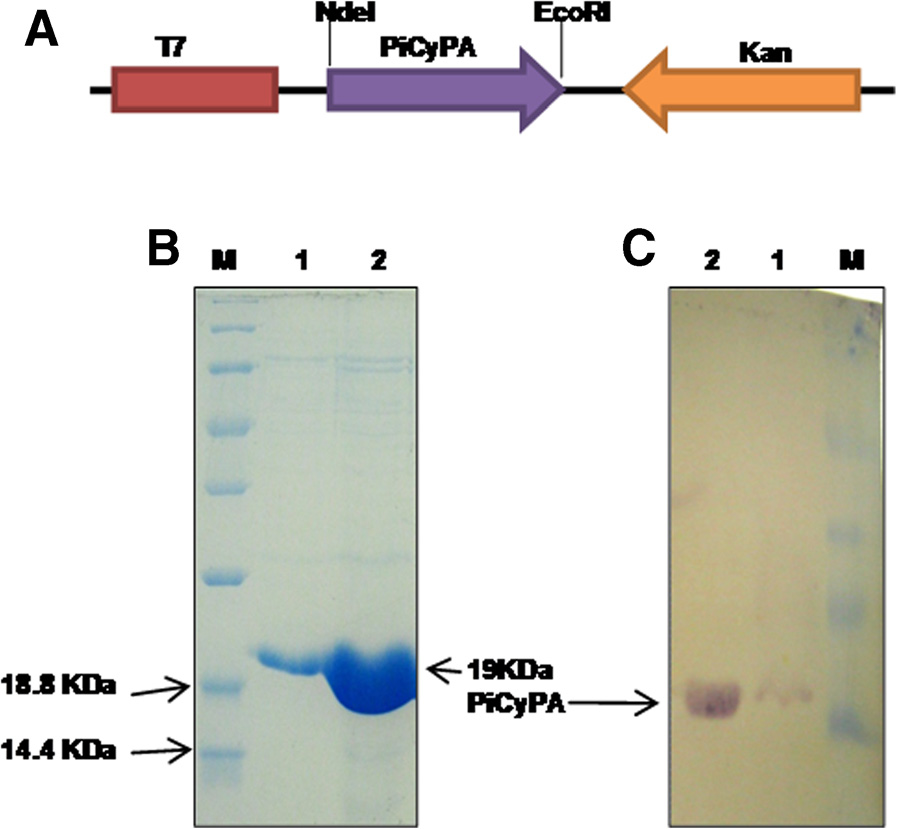
**Cloning and characterization of PiCyPA.** The CypA-pET28a construct, regulated by T7 promoter **(A)**. The determination of PiCyPA protein expression via commassie blue-stained gel and Western blot of purified PiCyPA: lane 1, molecular weight marker; lane 2 and lane 3, purified 50 ng and 100 ng of PiCyPA in eluted 200 mM as well as 500 mM imidazole fractions, respectively **(B**-**C)**.

The *PiCyPA* gene was PCR amplified from pBSK-*PiCyPA* construct using forward: 5` CTCGAGCATATGTCCCAGCCCAACGTCTACTTTG 3` and reverse: 5`-GAATTCTTAGACAGTGCCAGACGCAGTAATCTTG 3` primers and resulted PCR product of 535 bp size was cloned into the pGEMT-easy vector (Promega) and then to pET-28a vector (Novagen) by means of NdeI and EcoRI restriction sites, producing the pET-28a-PiCyPA construct (Figure 3A). The *PiCyPA* gene was expressed in *E. coli*, inserting a six-histidine tag onto its C terminus. The roughly 19–kDa PiCyPA protein was purified close to homogeneity and afterward it was verified by SDS-PAGE analysis (Figure 3B). The distinctiveness of the purified PiCyPA protein was further established by western blot study employing anti-His antibody (Figure 3C). The obtained purified preparation was used to assay the enzyme activity.

### Enzymatic activity of the purified PiCyPA protein

**Figure 4:**
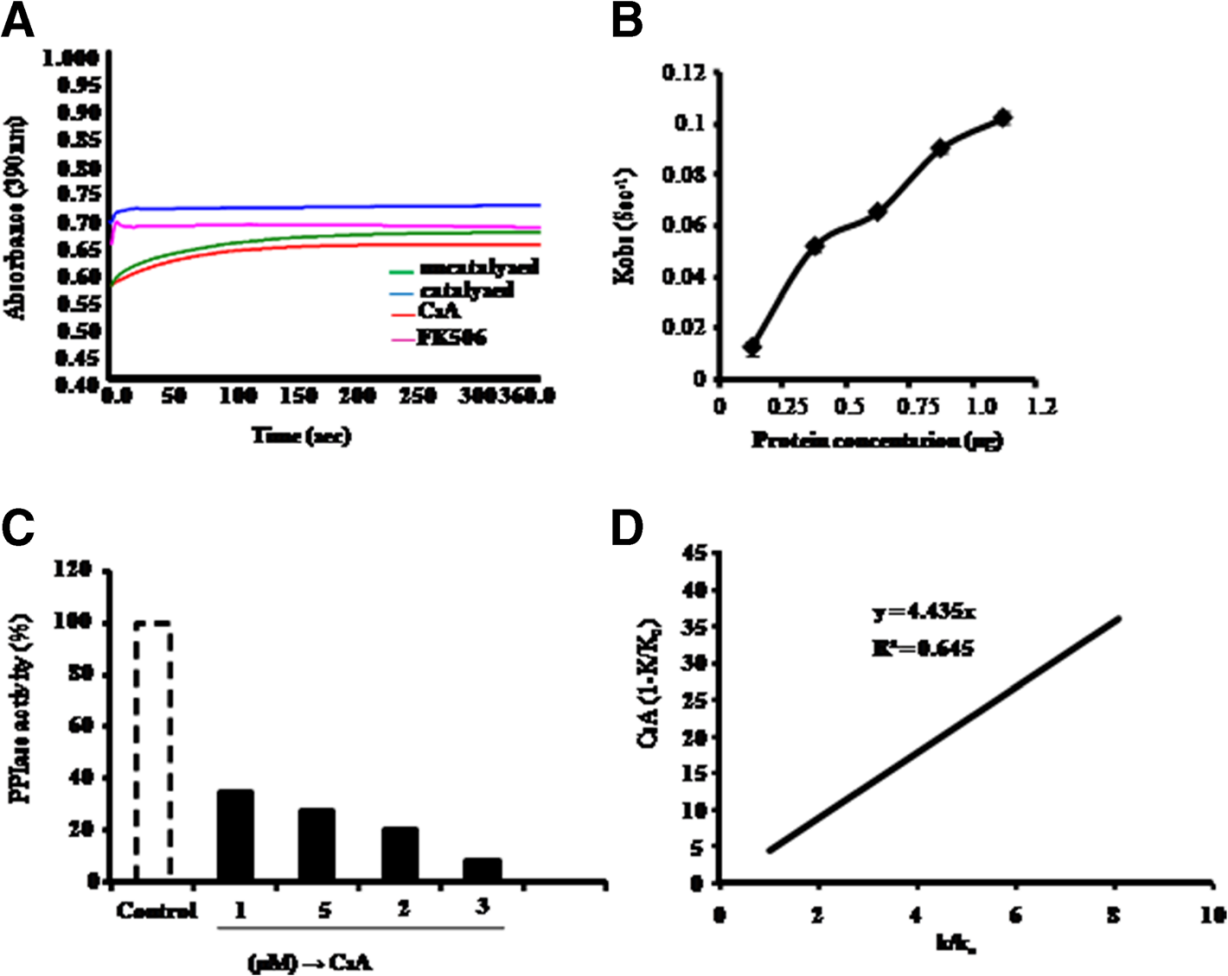
**Kinetics of purified PiCyPA cyclophilin.** It catalysed hydrolysis of N-succinyl-ala-ala-pro-phe-p-nitroanilidine (peptidyl prolyl *cis*-*trans* isomerase or PPIase activity) **(A)**. The dependence of rate constant on the cyclophilin concentration **(B)**. Effect of cyclophilin inhibitor, CsA, on PPIase activity of purified cyclophilin. The data represent the PPIase activity as percent of uninhibited control **(C)**. Determination of inhibition constant of purified CyPA for CsA **(D)**. First order rate constant was analysed using GraFit 4.0 software. Inhibition constant (ki) for CsA was determined as gradient of the line of the best fit from a plot of [CsA]/(1-k/k_o_) against k/k_o_, where k is the rate constant at any given CsA concentration and k_o_ is the rate constant in the absence of CsA. The slope of the line represents the k_i_. Data represent the means SDs of three independent experiments (n = 3).

The purified PiCyPA exhibited PPIase enzymatic activity since the first order rate constant (0.104 s^-1^) in the presence of 1 μg of this protein was almost 10-fold higher than the first order rate constant (0.010 s^-1^) observed for the uncatalysed control (Figure 4A). Further, the first order rate constant in the presence of purified PiCyPA showed an increase with increase in the protein concentration (Figure 4B), thus, implying that the observed PPIase activity was specifically contributed by the PiCyPA. The addition of equal amount of BSA had no significant effect on first order rate constant as compared to the uncatalysed reaction (data not shown). To demonstrate that the purified protein is a true cyclophlin, the PPIase activity of PiCyPA (1 μg) was estimated in the presence of FK506 and CsA. It is evident that the PPIase activity of the purified PiCyPA was inhibited dramatically only in the presence of CsA. The presence of CsA at 3 μM concentration resulted in almost 90% decrease as compared to the uninhibited control (Figure C). Further, inhibition constant was determined in order to determine as to how potent the CsA is as an inhibitor of this PiCyPA. It was observed that the inhibition constant of CsA for inhibition of PiCyPA is 4.435 nM. (Figure 4D).

### Abiotic stress tolerance in *E. coli* transformed with *PiCyPA* gene

**Figure 5:**
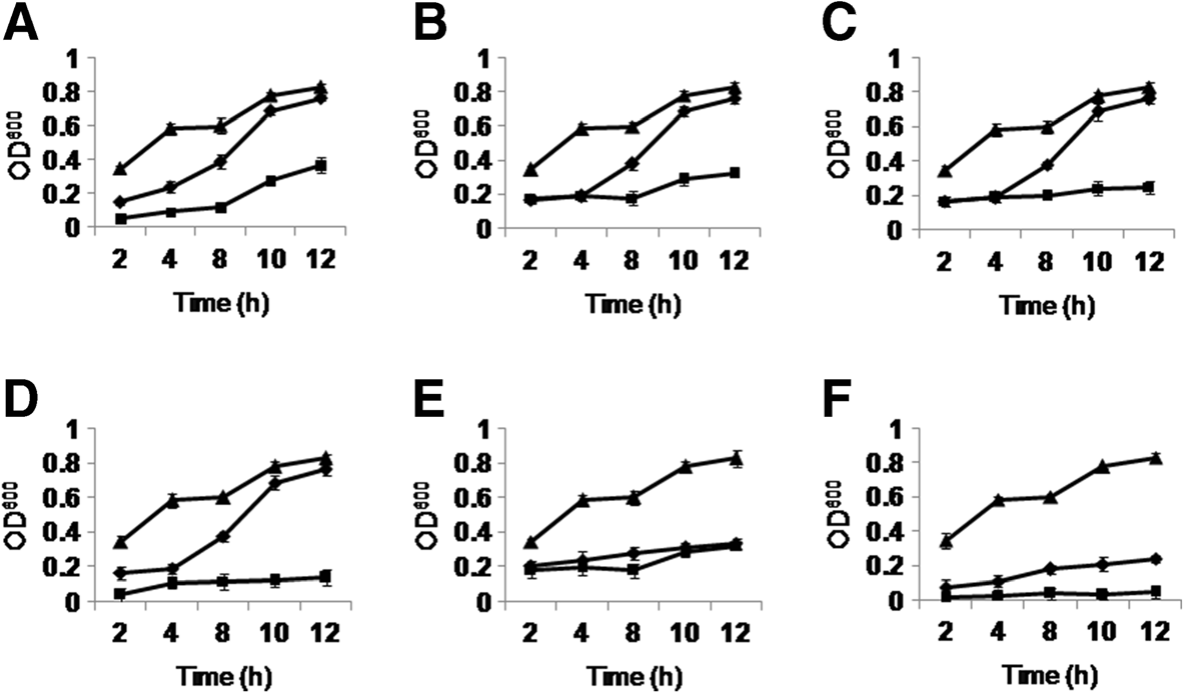
**The growth curves of empty pET28a and *CyPA***-**pET28a vector transformed *E. coli *under different abiotic stresses.** The growth curves of *CyPA*-pET28a transformed *E. coli* (BL21 codon plus) and empty pET28a vector transformed *E. coli* showed a gradual drop as a function of different time (h) intervals under different abiotic stresses such as salt (NaCl, 400 mM) **(A)**, cold **(B)**, heat **(C)**, cadmium chloride (CdCl_2_, 0.1 mM) **(D)**, cobalt chloride (CoCl_2_, 1 mM) **(E)** and hydrogen peroxide (H_2_O_2_, 0.4 mM) **(F)** as compared to no stressed (control). Data represent the means SDs of three independent experiments (n = 3). (*CyPA*-pET28a, No stress; *CyPA*-pET28a plus stress; Empty pET28a plus stress).

PiCyPA overexpression causes *E. coli* (DH5α) to tolerate abiotic stresses such as salinity, cold, heat, cadmium chloride (CdCl_2_), cobalt chloride (CoCl_2_) and hydrogen peroxide (H_2_O_2_). The assessment of the PiCypA capability to impart abiotic stress tolerance to bacteria was carried out qualitatively by estimating the OD_600_ of the bacterial culture as well as quantitatively by estimating turbidity in liquid LB culture medium. Three cultures (i) pET28a-*PiCyPA* containing *E. coli* cells plus No salt, (ii) empty pET28a containing *E. coli* cells plus 400 mM NaCl, (iii) pET28a-*PiCyPA* containing *E. coli* cells plus 400 mM NaCl were compared. It showed that an increase in OD of the *E. coli* cells harbouring pET28a- *PiCypA* was observed under described stress conditions at definite time intervals, although the empty pET28a whose OD was remained almost stagnant upon stress induction (Figure 5A-F).

## Discussion

Cyclophilins (CyP), generally conserved in all genera together with plants, are playing a part in diverse processes *viz*., cell division, transcriptional regulation, protein trafficking, cell signaling, pre-mRNA splicing, molecular chaperoning and stress tolerance [22,23]. Here, we identified a new cyclophilin gene (*PiCyPA*) (Accession No. GQ214003) from a root colonizing *P. indica* fungus [24]. Fungal stress signaling pathways have evolved rapidly in a niche-specific fashion that is independent of phylogeny and are relatively well conserved [25]. Hence, the stress response mechanisms reported in case of other fungi might also apply to *P. indica* and the genes responsible might also play similar role [22]. In this study, the PiCyPA encoded protein (Accession No. GQ214003) is highly specific by its PPIas activity and showing differential response against abiotic stresses. Introns are general characteristic of genes of higher eukaryotes. Alternative splicing is a mechanism which greatly enhances the biodiversity of proteins that genome of a particular organism can code for [26]. The genomic organization *PiCyPA* gene in *P. indica* genome has been identified via NCBI (http://www.ncbi.nlm.nih.gov), indicating 1304 bp *CyPA* gene, comprising 10 exons and 9 introns and upon excision of introns it was 535 bp (Figure 1A). The genome organization, biochemistry and molecular biology of the fungus in relative to its mode of action to provide tolerance against abiotic and biotic stress are indeed required to elucidate [27]. Further, a similar trend of genomic organization for *PiCyPA* gene was evident in PCR products (1304 bp and 535 bp fragments) with *P. indica* gDNA and cDNA (Figure 1B) and Southern profile (Figure 1C). Current data gives us the idea that analogous abiotic stress tolerance genes exist in both lower- and higher-organisms including plants, suggesting that common mechanisms for stress tolerance are emerging across the phylogenetic spectrum [26]. *PiCyPA*-like gene was indicating sequence similarities with CyP representatives of *L. bicolor*, *Homo sapiens*, *Arabidopsis thaliana*, yeast, rice and *E. coli* (Figure 2A). We have constructed rooted phylogenetic trees which were bootstrapped with 1000 replicates using MEGA version 5 [28]. It demonstrated that *P. indica CyPA* is closely related to human *CyP* in respect to high bootstrap value (Figure 2B).

The heterologous expression was observed in *E. coli* and *S. cerevisiae* for rice cyclophilin gene (OsCyP2) conferring tolerance to multiple abiotic stresses [29]. Here, *P. indica* cyclophilin gene (*PiCyPA*) gene was expressed in *E. coli* and its protein was purified and confirmed (Figure 3A-C). The evaluation of the rate constants of *cis*-*trans* isomerization of the peptide free in solution and bound to cyclophilin was categorized earlier. Dissociation of the Michaelis complexes were in similar magnitude as the isomerization rates on the enzymes, contributed steady state parameters [30]. The purified PiCyPA exhibited PPIase enzymatic activity which was 10-fold higher than the first order rate constant in uncatalysed control (Figure 4A), it increases with increase in the PiCyPA protein concentration (Figure 4B). The inhibition constant (4.435 nM.) of CsA observed for the recombinant PiCyPA is comparable to the inhibition constants observed for other cyclophilins such as fava bean (3.9 nM) [31] and maize (6 nM) [32], but lower than that reported for the human cyclophilins (2–200 nM) [33], thus signifying the variability in sensitivity of different CyPs to CsA. The difference in sensitivity of different CyPs to CsA is in accordance with the earlier reports [34], which demonstrated that PPIase activity of two maize CyPs, TLP40 and TLP20, showed differential sensitivity to inhibition by CsA. It is evident that the PPIase activity of the purified PiCyPA was inhibited dramatically only in the presence of CsA (Figure 4C). The presence of CsA at 3 μM concentration resulted in almost 90% decrease as compared to the uninhibited control (Figure 4C). Further, inhibition constant was determined in order to determine as to how potent the CsA, an inhibitor of this PiCyPA. It was observed that the inhibition constant of CsA for inhibition of PiCyPA is 4.435 nM. (Figure 4D).

The role of cyclophilin in abiotic stress tolerance has been well documented in various systems including prokaryotes as well as eukaryotes [29,35-37]. The analysis of transgene also revealed that bacteria expressing the PiCyPA protein could perform much better in multiple abiotic stress conditions as compared to the control. The result was supported by qualitative as well as quantitative data. In the qualitative analysis, the OD of the control samples remained unchanged while the OD of the PiCyPA transgenic bacteria continued to rise. The quantitative estimation analysis in salinity stress condition showed significantly higher growth of PiCyPA expressing bacteria as compared to wild type. These data suggest that the PiCyPA protein is likely to be a part of the general cellular stress response to multiple abiotic stresses which is conserved in prokaryotes, fungus and plants [29,37-39]. The pET28a-PiCyPA transformed bacterial cells showed enhanced tolerance to multiple abiotic stress as compared to empty pET28a vector containing bacterial cells (Figure 5A-F). The present investigation relies on the piece of evidence that the multifunctional PiCyPA embraces assure as a key candidate gene for enhancing abiotic stress tolerance to transgenic plants and recombinant proteins production.

## Conclusions

Our findings ascertain that *P. indica* cyclophilin (PiCyPA) proteins do take a part in a cellular response to mediate various physiological and molecular processes under abiotic stress condition in *E. coil*. The expression of PiCyPA protein enhanced the transgenic bacterial cells tolerance against wide range of abiotic stresses revealed a positive correlation between *PiCyPA* and stress response. Presented finding rely on the fact that PiCyPA might be a key player to confer stress tolerance and therefore it may potential hand round as a 'suitable manager for raising transgenic plants tolerance to multiple abiotic stress. Consequently, this study also offers the implication of PiCypA in recombinant proteins production. Further, extended stress conditions do not affect the PiCyPA protein conformation and might be detrimental for protein quality and quantity improvement. Additionally, PiCyPA protein via performing a module in stress signalling net work possibly aid in understanding physiological and molecular processes in relation to stress mitigation. The underlying stress tolerance mechanism in *E. coli* targeted by *P. indica* cyclophilin (PiCyPA) has not been worked out in plants in best of our knowlege. Further, fine tune insight into the mechanism of stress tolerance potentially mediating via PiCyPA in plants is currently in progress. This novel abiotic stress responsive PiCyPA protein and its future exploitation are indeed required in crop improvements to sustain the productivity under adverse environmental conditions.

## Methods

### Identification of CyPA gene of *P. indica*

Cyclophylin A-like protein (CyPA) (accession number GQ214003) was carefully chosen from cDNA library and its genomic organization CyPA was studied via NCBI (http://www.ncbi.nlm.nih.gov). A cDNA library was constructed from 5 μg of poly (A) + RNA (isolated from *P. indica* grown in 0.6 M NaCl) in Uni-Zap XR vector using Zap-cDNA synthesis kit (Stratagene, La Jolla, CA). Using an *in vivo* excision system the library was converted to phagemids and transferred in SOLR *E. coli* cells. Plasmids, pBluescript SK- (pBSK) containing cDNA inserts were mass-excised from phage stock of the *P. indica* cDNA library using ExAssist helper phage and propagated in SOLR *E. coli* cells. The cDNAs of *P. indica* were cloned downstream of the *lac* promoter of pBSK plasmids thus allowing the expression of recombinant proteins upon isopropyl-β-D-thiogalactopyranoside (IPTG) induction. Over one million *E. coli* recombinant cells from the same bacterial culture were plated on LB agar containing 50 μg/ml Kanamycin, 50 μg/ml Ampicillin, 1 mM IPTG and 0.6 M NaCl (concentration not permissible for the host bacterial growth). As a control the cells were also grown in the above medium with no extra salt included. The plates were incubated at 37°C for 12 to 16 hrs as described earlier [40]. 36 bacterial colonies were able to grow on LB plates supplemented with 1 mM IPTG and 0.6 M NaCl at 37°C. These colonies were plated on the same medium to confirm their abilities to tolerate high concentration of salt (0.6 M NaCl). *E. coli* cells with pBSK vector were used as negative controls. To further confirm the effective contribution of fungal cDNAs to bacterial NaCl survival and to exclude any association of the observed phenotype with unpredictable chromosomal mutations, the plasmids were purified from these over-expressing colonies in *E. coli* SOLR strain and reintroduced into a different *E. coli* strain (DH5α) and re-plated in LB plates containing IPTG and 0.6 M NaCl. Plasmids from these 36 positive colonies (*E.coli* DH5α) were sequenced on both strands by the dideoxy chain termination method, using Sequenase program Version 2.0 (US Biochemicals, Cleveland, OH, USA). The clones of the expression library were found to be in frame with the *LacZalfa* gene, which is driving expression in pBSK plasmid. Sequences were compared to GenBank database using BLAST N or BLAST X software (http://blast.ncbi.nlm.nih.gov/). One of the clone, cyclophylin (accession number GQ214003), was selected for further studies.

### Genomic Organization, isolation and substantiation of *PiCyPA*

The information of genomic organization of *PiCyPA* has been taken from contigs sequences of *Piriformospora indica* submitted in Pubmed (http://www.ncbi.nlm.nih.gov/pubmed/). Genomic organization of PiCyPA has been validated by PCR with gDNA and cDNA using PCR primers (forward: 5` CTCGAGCATATGTCCCAGCCCAACGTCTACTTTG 3` and reverse: 5`-GAATTCTTAGACAGTGCCAGACGCAGTAATCTTG 3`), respectively. Further *PiCyPA* gene copy numbers have been validated by southern blotting via standard protocol.

### Phylogenetic analysis

Protein sequences of cyclophilins (CyP) from different representative organisms were downloaded from NCBI database. These CyP sequences were aligned by means of ClustalW using default parameters. Phylogenetic analysis was accomplished using MEGA version 5 [41]. To build phylogenies, neighbour joining method [42] was employed and bootstrap analysis was performed by means of 1000 replicates. The phylogram was rooted using distantly related CyP sequences of different organisms.

### Cloning of PiCyPA gene into the pET28a expression vector

The entire sequence of cyclophilin gene, cloned into pBSK vector was amplified by using PCR primers (forward: 5` CTCGAGCATATGTCCCAGCCCAACGTCTACTTTG 3` and reverse: 5`-GAATTCTTAGACAGTGCCAGACGCAGTAATCTTG 3`). The PCR product was subsequently cloned into the pGEMT-easy vector (Promega) and it was sequenced using the T7 and SP6 primers respectively. After that it was subcloned into the pET-28a vector (Novagen) using the NdeI and EcoRI restriction sites to generate the pET-28a-PiCyp A construct for further characterization of cyclophilin protein.

### Protein expression and Purification

pET28a-PiCyPA construct was transformed into *E. coli* BL21 (*DE3*) codon plus cells. Transformed cells were grown in LB medium at 37°C with continuous shaking as 175 rpm. Culture was induced at OD_600_ ~ 0.8 using 0.5 mM IPTG at 18°C for overnight. Cells were harvested by centrifugation at 5000 g for 20 min and the protein was induced and purified using Ni-NTA (Qiagen, http://www.qiagen.com) resin and standard protocols. The protein was checked for purity by SDS-PAGE [10% (w/v) polyacrylamide gel] and commassie staining.

### Western blot analysis

The protein analysis was made by SDS-PAGE and transferred electrophoretically to nitrocellulose membrane by means of standard method. Subsequent to blocking, the membrane was developed with the suitable primary antibody (Penta-His; Qiagen) at defined period of 3 h at 27°C. The blot was then raised with the appropriate secondary antibody linked to alkaline phosphatase (SigmaAldrich, http://www.sigmaaldrich.com) and then developed via the standard method.

### Peptidyl prolyl *cis*-*trans* isomerase (PPIase) assay

PPIase activity was assayed at 15°C for 360 s in a coupled reaction with chymotrypsin as described earlier [42]. The one ml assay mixture contained 40 μM N-succinyl-ala ala-pro-phep- nitroanilidine as test peptide, assay buffer [50 mM HEPES (pH 8.0), 150 mM NaCl, 0.05% Triton X-100] and 30–50 μg of total proteins. The reaction was initiated by the addition of chymotrypsin (300 μg/ml) and the change in absorbance at 390 nm was monitored using a spectrophotometer (Perkin-Elmer Lambda Bio20) equipped with a Peltier temperature control system. The effect of FK506 and cyclosporine A (CsA), which are specific inhibitors of PPIase activity associated with FK506-binding proteins (FKBPs)- and cyclophilins was estimated by the addition of inhibitors to the assay mix 30 min before the start of the reaction, which followed by an incubation at 4°C. The PPIase activity was calculated as the product of the difference in the catalysed and uncatalysed first order rate constants (derived from the kinetics of the absorbance change at 390 nm) and the amount of substrate in each reaction [43].

### Growth of *E. coli* bacteria transformed with *PiCyPA* gene under abiotic stresses

The *E. coli* (BL21 codon plus cells) were transformed with PiCyp-pET28 and empty pET28a with the standard method. The transformed BL21 codon plus cells were first allowed to grow to log phase OD600 = 0.5. The equal amount of these cells were transferred to sterile culture tubes with 10 ml of LB medium containing 50 μg/ml kanamycin, 1 mM IPTG (final concentration) and 400 mM NaCl (final concentration). The cells were allowed to grow at 37°C and the growth was monitored by taking the OD600 at the interval of 2 h. For other stresses, the overnight grown BL21 codon plus cells in a LB medium were then cultured until the mid-log phase and then incubated it for an additional 2 h after 0.2 mM IPTG treatment with consistent shaking under different stresses like cold, heat, 0.2 mM CdCl_2_, 1 mM CoCl_2_, 0.4 mM H_2_O_2_.

## Abbreviations

cDNA: Complementary DNA; bp: Base pairs; PPIase: Peptidyl prolyl isomerase; CsA: Cyclosporin A; Nm: Nano molar; NCBI: National center for biotechnology information; gDNA: Genomic DNA; PCR: Polymerase chain reaction; kDa: Kilodaltons; SDS-PAGE: Sodium dodecyl sulfate polyacrylamide gel electrophoresis; His: Histidine; s-1: Per second; BSA: Bovine serum albumin; μg: micro gram; μM: micro molar; CdCl2: Cadmium chloride; CoCl2: Cobalt chloride; H2O2: Hydrogen peroxide; OD: Optical density; mRNA: Messenger RNA.

## Competing interests

The authors have declared that no competing interests exist.

## Authors’ contributions

DKT carried out the molecular study of cyclophilin A like protein from *P. indica* (PiCyPA) and participated in its genomic organization, identification, isolation, molecular characterization and sequence alignment, cloning into the pET28a expression vector, western blot analysis and its expression in *E.coli* ess. TD performed the prolyl *cis*-*trans* isomerase (PPIase) assay. MWA coordinated with the experiments on biotic stress tolerance in *E. coli* and performed data analysis, interpreted the results and drafted the manuscript. PS desined the enzyme kinetics assay, performed the analysis of data, and interpreted the results. NT conceived of the study and designed the experiments. All authors read and approved the final manuscript.
